# Bruxism and Sleep Disorders in Patients Diagnosed With Depressive Disorder and Anxiety Disorder Using Antidepressants

**DOI:** 10.1111/joor.13875

**Published:** 2024-10-09

**Authors:** Gizem Nur Pala Avan, Ali Erdoğan, Buket Cinemre, Burak Kulaksızoğlu, Özmen Metin

**Affiliations:** ^1^ Department of Psychiatry Akdeniz University Faculty of Medicine Antalya Turkey

**Keywords:** antidepressant, anxiety, bruxism, depression, sleep disorder

## Abstract

**Objective:**

To investigate the frequency of bruxism, factors associated with bruxism and sleep disorders in patients diagnosed with depressive disorder and anxiety disorder who use antidepressants.

**Methods:**

A total of 273 patients diagnosed with anxiety disorder or depression who had been using antidepressants for at least 1 month were included, along with 273 healthy control groups. The patient and control groups completed a sociodemographic data form, Epworth Daytime Sleepiness Scale (EDSS), Pittsburgh Sleep Quality Index (PSQI) and a bruxism questionnaire. Additionally, the clinician confirmed the diagnosis of bruxism through a clinical interview.

**Results:**

Bruxism was detected in 73.3% of the patient group and 28.2% of the control group (*p* < 0.001). The most commonly used antidepressants among patients were selective serotonin reuptake inhibitors (SSRIs) such as escitalopram and sertraline. Within the patient group, individuals with bruxism had higher family history rates of teeth grinding (*p* = 0.034), PSQI scores (*p* < 0.001) and EDSS scores (*p* < 0.001) compared to those without bruxism. Positive correlations were found between the presence of bruxism and PSQI (*p* < 0.001) scores as well as EDSS scores (*p* < 0.001) in both the patient group and all participants. Regression analysis conducted on the entire sample revealed that family history rates of teeth grinding (*p* < 0.001), antidepressant use (*p* < 0.001) and PSQI score (*p* = 0.004) were associated with bruxism.

**Conclusion:**

The findings from this study suggest that a majority of patients diagnosed with depressive or anxiety disorders may experience bruxism, particularly those using SSRI‐type antidepressants. Furthermore, individuals with bruxism may have poor sleep quality and excessive daytime sleepiness tendencies.

## 1 Introduction

Depression and anxiety disorders are common psychiatric illnesses that have a tendency to recur, respond well to treatment and can become chronic if left untreated, potentially leading to severe complications such as suicide. Antidepressants are widely used as pharmacological treatment for both depression and anxiety disorders [1].

Bruxism is currently defined as jaw muscle activity that can occur while awake or during sleep. Sleep bruxism and awake bruxism are two distinct forms of bruxism. Previously, bruxism was believed to be equivalent to nocturnal teeth grinding. This definition has modified as of 2018; sleep bruxism is now defined as masticatory muscle activity that occurs during sleep and can be either nonrhythmic (tonic) or rhythmic (phasic). In otherwise healthy people, it is not classified as a movement disorder or a sleep disorder. Awake bruxism is a term used to describe the repetitive or continuous movement of the lower jaw, involving tooth contact and/or bracing or thrusting of the mandible, during periods of wakefulness. Likewise, it is not a movement disorder that affects people who are otherwise healthy [2, 3]. This frequent health problem can occur at any stage of life, with a prevalence ranging from 9% to 31% in adults [4]. Although the exact nature of this widespread muscle disorder is not fully understood, it is believed to have a multifactorial etiology with potential effects on the central nervous system [5].

Psychosocial factors play an important role in the development of bruxism, particularly stress being considered as its main triggering factor [6]. Genetic factors may also contribute significantly to the aetiology of bruxism, as studies have shown that 21%–50% of individuals with sleep bruxism have a family history of the condition [7]. Additionally, psychotropic drugs, such as selective serotonin reuptake inhibitors (SSRIs) and serotonin noradrenaline reuptake inhibitors (SNRIs), have been implicated in causing bruxism [8]. Mental illnesses are also known to be associated with bruxism, with higher prevalence rates observed in individuals with recurring episodes of mental illness [9].

Furthermore, there seems to be a strong relation between bruxism and sleep disturbances. Individuals with bruxism often experience lower sleep quality and excessive daytime sleepiness, which negatively impacts their overall quality of life [10]. The existing literature provides evidence for an interrelationship between bruxism and psychopathology, highlighting its significance as a clinical problem in psychiatric practice and underscoring the need for further investigation.

Our study aims to compare patients using antidepressants with healthy controls in terms of the prevalence of bruxism and sleep disorders. We also seek to understand the relationship between bruxism and sleep disorders, as well as identify the factors that influence bruxism. Our H1 hypothesis is that the prevalence of bruxism is greater in individuals who use antidepressants compared to healthy controls and patients diagnosed with bruxism are more prone to experiencing sleep disturbance and excessive daytime sleep. Bruxism is an often overlooked problem among clinicians. Thus, our study might contribute to the existing literature regarding the prevalence of bruxism and highlight associated factors with this clinical condition.

## 2 Methods

### 2.1 Sample

Our study involved 273 patients and 273 healthy individuals who shared similar sociodemographic characteristics with the patients. Our study is a cross‐sectional study. The patients were outpatients who were being monitored at the Akdeniz University Faculty of Medicine Department of Psychiatry between July 2022 and March 2023, for either depressive disorder or anxiety disorder based on DSM‐5 criteria and were using antidepressants. The study included patients who agreed to participate and was designed as a cross‐sectional study.

The sample selection was made among the patients who applied to the outpatient clinic between the specified dates, met the inclusion criteria and agreed to participate in the study. The study was terminated upon reaching the desired sample size calculated in the power analysis. The 485 patients who either did not match the inclusion criteria or met the criteria but declined to participate were not enrolled in the study. A random sample approach was used to choose healthy controls comprising persons of similar age and gender to the patients, without a psychiatric diagnosis or drug use, and without any physical disease. A medical physical examination, history and medical records were used to assess the presence of a physical illness.

Considering the results obtained from a reference study [8], the minimum clinically significant difference in the incidence of bruxism between groups using antidepressants and those not using antidepressants was determined to be 10% (25% vs 15%). With an error margin of 0.05 and a power of 0.80, it was calculated that a minimum total sample size of 546 participants (273 per group) was required for this study. The G*Power 3.1.9.2 program was utilised to estimate the sample size. The inclusion criteria for patients included being over 18 years old, having a diagnosis of depressive disorder or anxiety disorder and exclusively using antidepressants for at least 1 month. Patients with a history of neurological disease, mental retardation, an additional psychiatric diagnosis other than depression and anxiety, substance or alcohol use disorders and using mood stabilisers/anxiolytics/antipsychotics other than antidepressants were excluded from the study.

All participants underwent various assessments including Sociodemographic Data Form, Structured Clinical Interview for DSM‐5 Axis I Disorders (SCID‐5) [11], Epworth Daytime Sleepiness Scale (EDSS) [12], Pittsburgh Sleep Quality Scale (PSQI) [13], as well as the Bruxism Questionnaire [14]. The Bruxism Questionnaire is a 6‐question survey developed based on the findings of the study conducted by Pintado et al. in 1997. According to the survey, individuals who responded ‘Yes’ to at least two of the questions were considered to have bruxism. Additionally, formal confirmation of bruxism diagnosis was obtained during psychiatric examinations conducted by the researcher.

#### 2.1.1 Sociodemographic Data Form

It is a semi‐structured data collection tool developed by researchers to assist in the collection of sociodemographic and clinical data, in accordance with the scope of the study.

#### 2.1.2 SCID‐5

It is a structured clinical interview scale developed by First and colleagues as a tool for clinical diagnosis, which can be administered by clinicians or mental health professionals familiar with the DSM‐5 diagnostic classification.

#### 2.1.3 EDSS

Developed by Johns in 1991, this scale is simple and self‐report based. This scale aims to measure the general level of daytime sleepiness and to assess the chance of falling asleep or dozing off in eight different daily life situations.

#### 2.1.4 PSQI

PSQI is developed in 1988 by Buysse et al. as an assessment tool to distinguish individuals with sleep disorders from healthy individuals, to determine the quality of sleep and to detect sleep problems. The scale consists of 24 questions in total. It comprises 18 scored questions distributed among seven categories. These categories include sleep duration, sleep latency, subjective sleep quality, sleep disturbance, habitual sleep activity, sleep medication use and daytime dysfunction. Each component is evaluated on a 0–3 point Likert scale. The total score of seven components gives the overall score of the scale which ranges from 0 to 21. The cut‐off score of the scale is 5 and scores of 5 and above indicate poor sleep quality.

The study received written approval from the Akdeniz University Faculty of Medicine Clinical Research Ethics Committee with decision number 451 on 20 July 2022. Participants provided written informed consent indicating their voluntary participation in the study. All stages of this study were carried out in accordance with the rules of the Helsinki Declaration.

### 2.2 Statistical Analysis

The data were analysed using IBM SPSS Statistics 18 Copyright SPSS Inc. 1989, the 2010 Software. The normality of continuous variables was assessed using the Kolmogorov–Smirnov test. Categorical variables in the study are reported as frequency (*n*) and percentage (%), while continuous variables are presented as median (IQR: 25–75) values. For the analysis of categorical variables, Pearson chi‐square, Fisher exact test and Fisher Freeman Halton exact test were employed, with post hoc Bonferroni correction applied. Since normal distribution was not observed in two independent group analyses, the Mann–Whitney U test was utilised. Spearman correlation analysis was conducted to explore relationships between continuous variables. Univariate and multivariate logistic regression analyses were performed to identify independent risk factors associated with dependent variables, wherein variables with *p* < 0.05 in univariate analyses were included in the multivariate model. The results obtained are presented as odds ratio (OR) and 95% confidence intervals. In this study, statistical significance level was set at *p* < 0.05.

## 3 Results

In our study, the median ages were 42 and 40 years in the patient and control groups, respectively (*p* = 0.603). Fifty‐five percent of the patients had depressive disorder, whereas the remaining 45% were diagnosed as having an anxiety disorder. SSRIs were the most frequently used antidepressants (*n* = 226) followed by escitalopram (*n* = 89), sertraline (*n* = 76), fluoxetine (*n* = 33), venlafaxine (*n* = 30), paroxetine (*n* = 28), duloxetine (*n* = 11), mirtazapine (*n* = 2), bupropion (*n* = 2) and vortioxetine (*n* = 2). Other sociodemographic and clinical characteristics of the study groups are summarised in Table 1.

**TABLE 1:** Comparison of sociodemographic and clinical characteristics of the study groups.

		Patient group (*n* = 273)		Healthy controls group (*n* = 273)		*p*
		*n*	%	*n*	%	
Sex	Male	69	25.3	69	25.3	> 0.999
	Female	204	74.7	204	74.7	
Marital status	Married	174	63.7	178	65.2	0.721
	Single	99	36.3	95	34.8	
Level of education	Primary school	69	25.3	41	15.0	0.005
	Secondary school	10	3.7	21	7.7	
	Gymnasium	94	34.4	91	33.3	
	University	100	36.6	120	44.0	
Employment status	Employed	124	45.4	184	67.4	< 0.001
	Unemployed	149	54.6	89	32.6	
Smoking	Yes	75	27.5	118	43.2	< 0.001
	No	198	72.5	155	56.8	
History of COVID	Yes	94	34.4	111	40.7	0.133
	No	179	65.6	162	59.3	
Family history of teeth Grinding	Yes	95	34.8	143	52.4	< 0.001
	No	178	65.2	130	47.6	
Sleep disturbance	Yes	128	46.9	78	28.6	< 0.001
	No	145	53.1	195	71.4	
Bruxism	Yes	200	73.3	77	28.2	< 0.001
	No	73	26.7	196	71.8	
Antidepressant use	Yes	273	100.0			< 0.001
	No	0	0.0			
Age (year) median (IQR)		42 (29–53)		40 (38–43)		0.603
Duration of antidepressant use (month) (median) (IQR)		5 (3–12)				
Pittsburgh Sleep Quality Index (median) (IQR)		7 (4–10)		3 (2–6)		< 0.001
Epworth Sleepiness Scale (median) (IQR)		6 (4–10)		5 (2–10)		0.015

The patient group was divided into two groups: those with and without bruxism, and the comparison of these two groups is summarised in Table 2.

**TABLE 2:** Comparison of individuals with and without bruxism in the patient group.

		Patients without bruxism (*n* = 73)		Patients with bruxism (*n* = 200)		*p*
		*n*	%	*n*	%	
Sex	Male	21	28.8	48	24.0	0.422
	Female	52	71.2	152	76.0	
Marital status	Married	47	64.4	127	63.5	0.893
	Single	26	35.6	73	36.5	
Level of education	Primary school	14	19.2	55	27.5	0.003
	Secondary school	0	0.0	1	5.0	
	Gymnasium	37	50.7	57	28.5	
	University	22	30.1	78	39.0	
Employment status	Employed	32	43.8	92	46.0	0.751
	Unemployed	41	56.2	108	54.0	
Smoking	Yes	22	30.1	53	26.5	0.551
	No	51	69.9	147	73.5	
Family history of teeth Grinding	Yes	18	24.7	77	38.5	0.034
	No	55	75.3	123	61.5	
Sleep disturbance	Yes	24	32.9	104	52.0	0.005
	No	49	67.1	96	48.0	
Age (year) median (IQR)		44 (31–53)		41 (28–52)		0.372
Pittsburgh Sleep Quality Index (median) (IQR)		4 (2–7)		7.5 (5–10)		< 0.001
Epworth Sleepiness Scale (median) (IQR)		5 (3–6)		8 (4–11)		< 0.001

In the healthy control group, those with bruxism (*n* = 77) and those without (*n* = 196) were also compared. Among healthy controls, the smoking rate in those with bruxism (66.2%) was higher than the smoking rate in those without bruxism (34.2%) (*p* < 0.001). Similarly, the rate of those with a family history of teeth grinding in those with bruxism (76.6%) was significantly higher than the rate of family teeth grinding in those without bruxism (42.9%) (*p* < 0.001). In healthy controls, the PSQI median score was significantly higher in subjects with bruxism than those without bruxism (5 (3–7) and 3 (2–5), respectively) (*p* < 0.001). The EDSS median score was also higher (6 (3–10)) in those with bruxism compared to those without bruxism (5 (1–9)), (*p* = 0.032).

In the patient group, the presence of bruxism showed significant positive correlations with PSQI (*r* = 0.299, *p* < 0.001) and EDSS (*r* = 0.282, *p* < 0.001) scores. The presence of bruxism showed significant positive correlations with PSQI (*r* = 0.399, *p* < 0.001) and EDSS (*r* = 0.222, *p* < 0.001) scores among all the participants.

In the entire sample, factors that independently affected the presence of bruxism were evaluated by univariate and multivariate logistic regression analysis. Factors with *p* < 0.05 in univariate analysis were included in the multivariate model. Accordingly, a family history of teeth grinding (OR: 2.432; 95% CI: 1.546–3.826; *p* < 0.001), antidepressant use (OR: 5.928; 95% CI: 3.708–9.478; *p* < 0.001) and a higher PSQI score (OR: 1.113; 95% CI: 1.034–1.198; *p* = 0.004) predicted the presence of bruxism.

Factors that independently affected the presence of bruxism in the patient group were evaluated by univariate and multivariate logistic regression analysis. Factors with *p* < 0.05 in univariate analysis were included in the multivariate model. Accordingly, having an education level at high school and above (OR: 0.454; 95% CI: 0.216–0.954; *p* = 0.037), PSQI score (OR: 1.170; 95% CI: 1.051–1.302; *p* = 0.004) and EDSS score (OR: 1.122; 95% CI: 1.039–1.212; *p* = 0.003) predicted the presence of bruxism.

## 4 Discussion

Our study revealed that the rate of bruxism in patients using antidepressants was 73.3%, significantly higher than the bruxism rate in the healthy control group (28.2%). The prevalence of bruxism in adults in the general population is reported to be between 9% and 31% [4]. A study conducted in Germany with 191 participants reported a bruxism rate of 24.7% [15]. Another study focusing on adults in the Netherlands showed a rate of 5.0% for wakefulness bruxism and 16.5% for sleep bruxism [16]. Our study detected bruxism in approximately one third (28.2%) of the healthy control group, consistent with existing literature, which together highlights it as a public health concern.

The findings in our study also showed that three‐quarters of patients using antidepressants had bruxism. A cross‐sectional study reported a higher prevalence of bruxism (24.3%) among patients using serotonergic antidepressants compared to the control group (15.3%) and the average time of onset of bruxism after the initiation of the antidepressant treatment was 2. 85 months [8]. Evidence suggests that both antidepressants and psychostimulants can trigger bruxism. A possible explanation is the indirect effect on dopaminergic activity of the long‐term use of these drugs [17]. A comprehensive analysis conducted on a total of 1688 patients identified antidepressant use as a potential determinant for developing bruxism [18]. Regression analysis performed during our study indicated that independent usage of antidepressants predicted bruxism, further highlighting the association between antidepressant treatment and bruxism. Furthermore, the frequency of bruxism detected in our patient group using antidepressant treatment was significantly high, possibly making it the highest rate reported in the literature. The median duration of antidepressant use among patients was 5 months, suggesting that a significant number of long‐term antidepressant users may experience bruxism. Thus, clinicians should consider evaluating and questioning bruxism within this patient group during follow‐up assessments.

In our study, night‐time sleep disturbance and daytime sleepiness were significantly more frequent in patients using antidepressants and having bruxism than those without bruxism. Similarly, a positive relationship between sleep bruxism and night‐time insomnia was observed in a study with 1042 people who were evaluated with polysomnography [19]. It has been reported that, in addition to sleep bruxism, wakefulness bruxism may also be associated with sleep disorders. In a cross‐sectional study involving 183 Brazilian dental students aged 17–46 years, both sleep bruxism and wakefulness bruxism were reported to be associated with poor sleep quality [20]. In a local study conducted in our country, 100 individuals with bruxism and 100 healthy control groups were evaluated, and the PSQI score was found to be significantly higher in those with bruxism [21]. However, there is also contradicting evidence, such as the findings of a study by Chattrattrai et al. [22] conveying that subjectively reported sleep bruxism was associated with insomnia, but not the sleep bruxism confirmed by polysomnography. In our study, bruxism was found to be associated with high school and above education level, PSQI score and EDSS score in the patient group. This finding may also suggest a significant relationship between bruxism and sleep disorders. Bruxism causes pain particularly affecting teeth or jaw muscles, thus potentially causing insomnia.

In our study, patients with bruxism using antidepressants had more insomnia and excessive daytime sleepiness than those without bruxism. Likewise, in healthy controls, night‐time sleep quality was found to be worse, and excessive daytime sleepiness was found to be more prevalent in those with bruxism than in those without bruxism. Evidence in the literature shows a significant positive correlation between daytime sleepiness and bruxism [23]. In a study conducted with 74 participants with possible sleep bruxism, 78.4% of the patients who underwent a one‐night polysomnography were diagnosed with sleep bruxism. EDSS scores of patients with sleep bruxism were found to be significantly higher than those without bruxism [24]. However, there are also some studies that do not support this association [25]. In our study, bruxism was found to be associated with excessive daytime sleepiness in patients using antidepressants. This finding may suggest that bruxism disrupts people's sleep quality at night, possibly causing excessive sleepiness during the day. Considering that three quarters of patients using antidepressants have bruxism, excessive daytime sleepiness may be an important issue in these patients, causing serious problems such as being late for work or even not being able to go to work, home accidents and traffic accidents. Therefore, patients using antidepressants should be closely monitored for bruxism and excessive daytime sleepiness.

Another important finding of our study is that the history of teeth grinding in the families of those with bruxism was significantly higher than in those without bruxism. Evidence shows that bruxism may be genetically inherited [26], which is consistent with our findings in the study. However, multicentre genetic studies of bruxism are needed for a better understanding of genetic aetiology of this movement disorder.

Our study is one of the limited number of studies in the literature that evaluates the frequency of bruxism, bruxism risk factors and the relationship between bruxism and sleep in patients diagnosed with anxiety and depression and using antidepressants. It is also one of the cross‐sectional studies with the largest sample size. Our study appears to be different from other studies due to its unique focus on the correlation between antidepressants and sleep disorders. Specifically, it uncovers the connection between SSRI group antidepressants, widely used globally, and bruxism, as well as the subsequent association between bruxism and sleep problems.

However, our study has some limitations. First, the study shows the results of a sample collected from a single centre. Another limitation is the lack of confirmation of bruxism by polysomnography.

## 5 Conclusion

The results of our study indicate that three quarters of the patients using antidepressants experience bruxism. Additionally, patients with bruxism are more prone to night‐time sleep disorders and excessive daytime sleepiness. Bruxism in the patients is correlated with a high school or higher education level, sleep disorder and excessive daytime sleepiness. When considering all participants, it can be concluded that bruxism is linked to antidepressant use, a family history of teeth grinding and sleep disorder. A higher prevalence of teeth grinding in the families of patients with bruxism suggests a potential genetic aspect to bruxism. Multicentre prospective studies evaluating all risk factors should be considered for future research about this common clinical problem.

## Ethics Statement

The Clinical Research Ethics Committee of Akdeniz University, Faculty of Medicine, approved the study (Decision number 451; Date: 20.07.2022), which was carried out following the rules of the Declaration of Helsinki.

## Conflicts of Interest

The authors declare no conflicts of interest.

### Peer Review

The peer review history for this article is available at https://www.webofscience.com/api/gateway/wos/peer‐review/10.1111/joor.13875.

## Data Availability

The data that support the findings of this study are available on request from the corresponding author. The data are not publicly available due to privacy or ethical restrictions.
